# Effect of Electrolytic Manganese Residue in Fly Ash-Based Cementitious Material: Hydration Behavior and Microstructure

**DOI:** 10.3390/ma14227047

**Published:** 2021-11-20

**Authors:** Yaguang Wang, Na Zhang, Yongyu Ren, Yingtang Xu, Xiaoming Liu

**Affiliations:** 1 State Key Laboratory of Advanced Metallurgy, School of Metallurgical and Ecological Engineering, University of Science and Technology Beijing, Beijing 100083, China; wangyg@xs.ustb.edu.cn (Y.W.); ren142517@163.com (Y.R.); yingtangx@163.com (Y.X.); 2 Beijing Key Laboratory of Materials Utilization of Nonmetallic Minerals and Solid Wastes, National Laboratory of Mineral Materials, School of Materials Science and Technology, China University of Geosciences, Beijing 100083, China

**Keywords:** electrolytic manganese residue, fly ash-based cementitious material, hydration behavior, pore structure, polymerization degree

## Abstract

Electrolytic manganese residue (EMR) is a solid waste with a main mineralogical composition of gypsum. It is generated in the production of metal manganese by the electrolysis process. In this research, EMR, fly ash, and clinker were blended to make fly ash-based cementitious material (FAC) to investigate the effect of EMR on strength properties, hydration behavior, microstructure, and environmental performance of FAC. XRD, TG, and SEM studied the hydration behavior of FAC. The pore structure and [SiO_4_] polymerization degree were characterized by MIP and ^29^Si NMR, respectively. The experimental results indicate that FAC shows excellent mechanical properties when the EMR dosage is 10%. Moderate content of sulfate provided by EMR can promote hydration reaction of FAC, and it shows a denser pore structure and higher [SiO_4_] polymerization degree in this case. Heavy metal ions derived from EMR can be adsorbed in the hydration products of FAC to obtain better environmental properties. This paper presents an AFt covering model for the case of excessive EMR in FAC, and it importantly provides theoretical support for the recycling of EMR in cementitious materials.

## 1. Introduction

Electrolytic manganese residue (EMR) is the main kind of solid waste generated in the production of metal manganese by the electrolysis process [1]. About 6–9 tons of EMR is produced with the production of per ton of manganese [2]. The prime hazardous substances in EMR are ammonia nitrogen (NH_3_-N) and manganese ion, leading to serious environmental pollution problems [3,4,5]. Although electrolytic manganese plants have used many different methods to dispose of EMR, the environmental pollution problem is still serious [6,7]. Therefore, rational recycling and utilization of EMR can alleviate the environmental problems caused by EMR and bring good environmental benefits to society. The electrolytic manganese enterprises will obtain good economic benefits as well [8].

Quartz and gypsum are the main mineral components in EMR. The difference between EMR and other slags is that EMR is rich in sulfate. According to the detection, the content of SO_3_ in EMR reaches 30% [9]. The sulfate in EMR exists in many forms, not limited to gypsum. For example, it can be found in ammonium sulfate and manganese sulfate [10]. In recent years, great progress has been achieved in the recycling of EMR. Preparation of building materials is a valid way for the recycling of EMR. Li et al. [11] found that if using EMR and fly ash to prepare a composite admixture, the strength of the cement system will be improved with the adding of calcined EMR. Moreover, EMR can be used as a raw material to produce sulfoaluminate-like cement [12] and sulfur concrete [13]. At present, the application of EMR in building materials is simply blended into cement and concrete. However, the effect of sulfate in EMR has not been considered in these researches [14,15,16].

Fly ash is one of the largest industrial solid wastes in the world. SiO_2_, Al_2_O_3_, Fe_2_O_3_, and CaO are the main chemical composition in fly ash [17,18]. It is a composite structure composed of crystal and amorphous phases under the microscope. Crystal phases include quartz, mullite, magnetite, etc. Amorphous phases include smooth spherical vitreous particles, small particles with irregular pore shapes, porous and irregular vitreous spheres [19]. Fly ash belongs to an artificial pozzolanic material that shows good cementitious activity in lime and gypsum solutions. Its cementitious activity mainly comes from the hydration of active SiO_2_ and active Al_2_O_3_ in the vitreous phases under certain alkaline conditions. Therefore, fly ash is commonly used as a cement admixture for its potential pozzolanic activity [20].

In previous studies [21,22,23], desulfurization gypsum or phosphogypsum has been applied to cement as a cement retarder because its main component is CaSO_4_·2H_2_O. It can react with calcium aluminate (C_3_A) in cement clinker to form ettringite and delay the setting time. Xu et al. [24] studied the effect of sulfate on the hydration characteristics of blast furnace slag. The results showed that a proper amount of sulfate could facilitate the reaction of blast furnace slag to produce C-S-H gel and AFt, which makes cementitious material’s internal structure more compact. Li et al. [25] studied the effect of gypsum on the working and mechanical properties of red mud and blast furnace slag-based cementitious materials. The results showed that different kinds of gypsum could reduce the fluidity of mortar, shorten the setting time, and improve the mechanical properties of mortar. It is worth noting that previous studies focused more on the effect of sulfate on the hydration of blast furnace slag by using desulfurized gypsum or phosphogypsum, which provides an effective reference for the utilization of EMR in cementitious materials. Although there is no effective way to use EMR, it contains a certain amount of sulfate. In addition, when electrolytic manganese slag is used as supplementary cementitious material, it contains many heavy metal salts, which must also be paid attention to.

In this work, EMR, fly ash, and clinker were used to prepare fly ash-based cementitious material (FAC) to investigate the effect of EMR with different dosages on the mechanical properties, hydration behavior, microstructure, and environmental performance of FAC. The hydration behavior was characterized by scanning electron microscopy (SEM), X-ray diffraction (XRD), and thermogravimetric analysis (TG). The pore structure and [SiO_4_] polymerization degree were analyzed by mercury intrusion porosimetry (MIP) and ^29^Si nuclear magnetic resonance (NMR), respectively. The environmental performance was analyzed by a heavy metal leaching test. This work helps to understand the real roles of EMR in cementitious materials. It is conducive to solving environmental problems through the effective utilization of EMR in construction and building materials.

## 2. Materials and Methods

**Figure 1:**
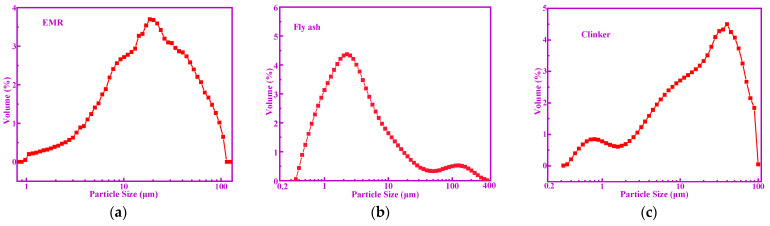
The particle size distribution of raw materials: (**a**) EMR, (**b**) fly ash, and (**c**) clinker.

EMR used in this work is the waste residue of an electrolytic manganese plant (Songtao Sanhe Manganese Industry Group Co., Ltd.) in Tongren, Guizhou province of China, and its color is black. Fly ash (FA) was also supplied by a power plant (Dalong power plant) from Guizhou province of China. The clinker was obtained from a cement plant (Tangshan Jidong Cement Co., Ltd) in Tangshan, Hebei province of China, the strength grade of which is 42.5. Standard sand was obtained from China ISO Sand Co., Ltd (Beijing, China). Table 1 shows the chemical composition of raw materials, in which EMR presents a high content of SO_3_ close to 30%. The specific surface areas of the FA, EMR, and clinker were 568.58 m^2^/kg, 610.73 m^2^/kg, and 454.27 m^2^/kg, respectively. The particle size distribution of raw materials is shown in Figure 1 (EMR: d_50_ = 15.24 μm; FA: d_50_ = 2.59 μm; Clinker: d_50_ = 17.93 μm).

**Figure 2:**
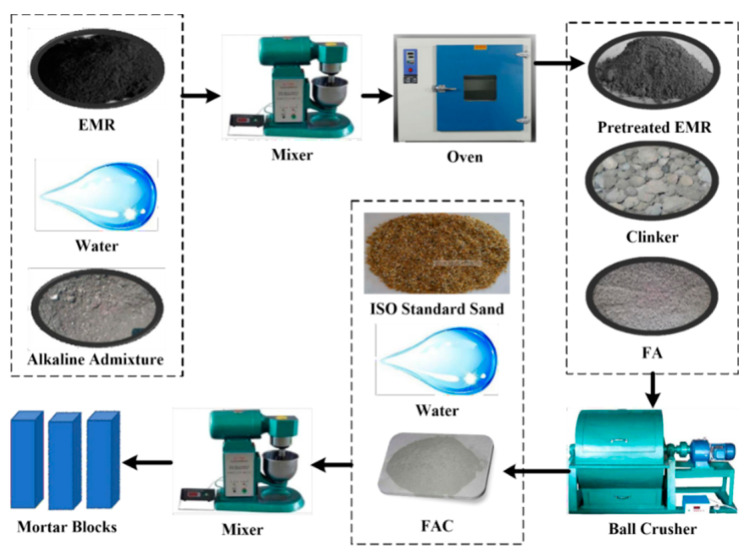
Preparation process of FAC and mortar blocks.

Previous studies have shown that it is necessary to pretreat EMR before it is used to reduce NH_3_-N content and solidify heavy metals [26]. In our experiment, a kind of alkaline admixture was used to pretreat EMR. The main component of this alkaline additive is carbide slag (The XRD spectrum of carbide slag is shown in Figure S1). Therefore, it can be used as lime to pretreat EMR. Firstly, EMR and alkaline admixture were mixed together at a 10:1 ratio, and then they were mixed well with water in a cement mortar mixer. Then they were dried in an oven until the mass no longer changed. The pretreated EMR, clinker, and FA were mixed as designed proportions are shown in Table 2. The raw materials were put into a ball crusher to mix and grind. After XRF (xrf-1800) analysis, the SO_3_ content in the resultant FAC can be obtained, and they are also listed in Table 2. It can be seen that the content of SO_3_ in the FAC samples increases obviously with the increasing dosage of EMR. An FBT-9 specific surface area tester obtained the fineness of FAC powder. The preparation process of FAC and mortar blocks is shown in Figure 2.

In order to investigate the effect of EMR on the mechanical properties of FAC, mortar blocks in size of 40 mm × 40 mm × 160 mm were prepared, in which the water/binder ratio was 0.5, and sand/binder ratio was 3. The mortar samples were kept in the curing box for maintenance for 24 h. The curing temperature was maintained at 25 ± 1 °C, and the humidity was 95 ± 1%. Then the mortar samples were released from the mold and placed in a maintenance pool, in which the water height was 2 cm taller than the upper surface of mortar samples. An automatic pressure testing machine obtained the mechanical properties of the FAC mortar samples.

In order to investigate the effect of EMR on the hydration characteristics and microstructure of FAC, paste samples in the size of 20 mm × 20 mm × 20 mm were prepared, in which the water/binder ratio was 0.35. The paste samples were cured for several days, and then they were taken out from the curing box for characterization. The hydration of paste samples was ended with absolute ethanol. After that, they were put in a vacuum drying box at 60 °C. The hardened paste after dehydrating with anhydrous ethanol and vacuum drying treatment can be used for MIP detection. These hardened pastes were ground for XRD, TG, and NMR analysis. The XRD data were recorded using a Bruker XRD-7000 diffractometer (Bruker, Beijing, China). The experimental conditions were 40 kV, 100 mA, Cu Target, and scanning speed of 4 °C/min. TG-DTG data were recorded by using a TGA 4000 thermogravimetric analyzer (PerkinElmer, Shanghai, China). The experimental conditions were N_2_, heating rate of 10 °C/min, and heating temperature range of 20 to 1000 °C. In the MIP analysis, the pore size distribution of the sample was measured by AutoPore IV 9500 automatic mercury injector (Micromeritics Instruments Corporation, Shanghai, China) at 25 °C and 60,000 psi (423,685 kpa). The high-pressure pore diameter analysis is a minimum 3 nm. The low-pressure analysis pressure range is 0.5–50 psia (3.45–310 kpa). The low-pressure aperture analysis range is 360–3.6 μm. The ^29^Si NMR spectroscopy was obtained by using Bruker ADVANCE III 600M spectrometer (Beijing, China) to study the [SiO_4_] polymerization degree of hydration products. The microstructure of FAC hydrated pastes was characterized by using a JEOL JSM-6701F scanning electron microscope (Beijing, China).

## 3. Results and Discussion

### 3.1. Effect of Electrolytic Manganese Residue on the Strength of FAC

**Figure 3:**
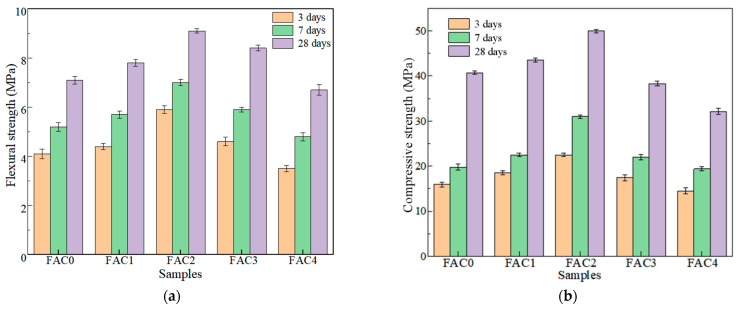
Strength of FAC mortars at different ages: (**a**) flexural strength and (**b**) compressive strength.

In this experiment, the flexural strength and compressive strength of FAC mortar samples at 3d, 7d, and 28d were tested, and the results are recorded in Figure 3. As shown from Figure 3a,b, with the increase of EMR dosage, the peak values of flexural strength and compressive strength appeared at the EMR dosage of 10%. Meanwhile, as maintenance time increased from 3d to 28d, the strength was also enhanced. The best 3d compressive strength of 22.5 MPa and 28d compressive strength of 50 MPa were obtained by FAC2 sample. The compressive strength of FAC2 can even reach the strength level of 42.5R ordinary Portland cement.

As the maintenance time increases, the strength of FAC mortar samples increases, but the strength of FAC3 and FAC4 is still below that of FAC2. The reason could be complicated. First, EMR contains a considerable number of inert components. When the EMR dosage is excessive, the increasing inert components will weaken the cementation properties of the FAC system to weaken the compactness and reduce the strength of the mortar specimen. Second, excessive gypsum in EMR can inhibit the hydration reaction and hinder the growth of AFt. Meanwhile, excessive gypsum could also lead to volume expansion and poor volume stability in the later period, resulting in small cracks in the sample and affecting the strength of FAC. Therefore, a proper amount of EMR can stimulate the formation of hydration products and improve the compactness of hardened FAC, but excessive EMR will reduce the strength of FAC.

Considering that the strength of FAC4 containing 20% EMR is lower than that of 42.5 ordinary Portland cement, the effect of 20% EMR on the hydration characteristics and microstructure of FAC was ignored in the following analysis.

### 3.2. Effect of Electrolytic Manganese Residue on Hydration Products of FAC

**Figure 4:**
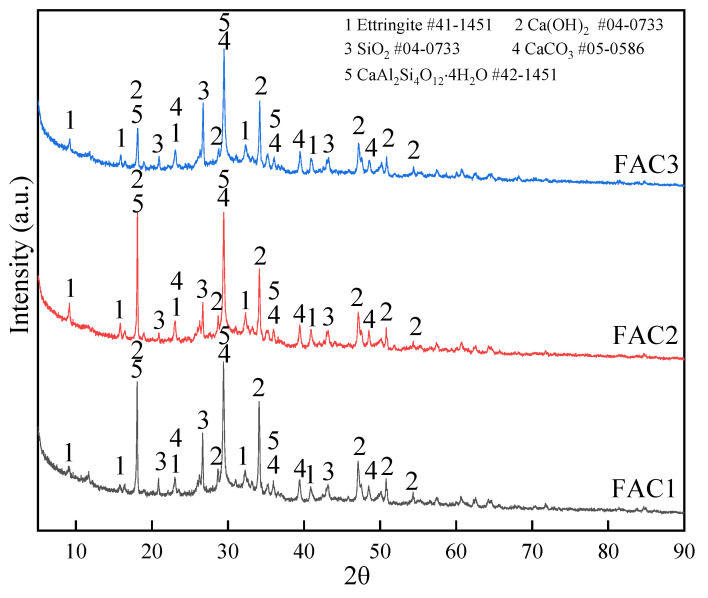
XRD patterns of FAC hydrated pastes at 28 days.

The XRD patterns of FAC paste samples (FAC1, FAC2, and FAC3) hydrated at 28d are shown in Figure 4. The main crystalline phases are ettringite (Ca_6_Al_2_(SO_4_)_3_(OH)_12_·26H_2_O), CaCO_3,_ and Ca(OH)_2_. Besides, there is also a kind of zeolite-like phase (CaAl_2_Si_4_O_12_·4H_2_O). The three XRD patterns present the dispersion peak between 23° and 37° indicative of amorphous compound C-A-S-H gels production. No diffraction peaks in the XRD spectrum are found for C-A-S-H gel and C-S-H gel because they are amorphous phases [27]. C-S-H gel and Ca(OH)_2_ are generally synthesized by the hydration of the clinker. The peak of CaCO_3_ is caused by partial carbonation of Ca(OH)_2_ and CaCO_3_ in the raw materials. The XRD spectrum of fly ash is shown in Figure S2. Moreover, the vitreous substances of fly ash can be dissolved when it is in an alkaline environment; the aluminum-oxygen and silicon-oxygen bonds in the vitreous substances are broken by the activation of Ca(OH)_2_ and gypsum to make more hydraulic substances like C-A-S-H gel and AFt [28]. The chemical reactions are presented as Schemes (1) to (5).

SiO_2_ + OH^−^ + H_2_O → [H_3_SiO_4_]^−^ (1)

AlO_2_^−^ + OH^−^ + H_2_O → [H_3_AlO_4_]^2−^ + [Al(OH)_6_]^3−^ (2)

[H_3_SiO_4_]^−^ + [H_3_AlO_4_]^2−^ + Ca^2+^ → C-A-S-H (3)

[H_3_SiO_4_]^−^ + [H_3_AlO_4_]^2−^ + Ca^2+^ → CaAl_2_Si_4_O_12_·4H_2_O (4)

[Al(OH)_6_]^3−^ + Ca^2+^ + SO_4_^2−^ + H_2_O → Ca_6_Al_2_(SO_4_)_3_(OH)_12_·26H_2_O (5)

It can be discovered from Figure 4 that the AFt peaks of the FAC2 sample are higher than the other two samples. Moreover, the peak of Ca(OH)_2_ in the FAC2 sample is lower than the other two samples. It indicates that the activation of fly ash in FAC2 was fully activated by Ca(OH)_2_ and the sulfate of EMR to produce more cementitious substances. In summary, there is an appropriate amount of EMR in the FAC2 sample, resulting in better strength properties. Insufficient EMR dosage cannot adequately stimulate the pozzolanic activity of fly ash at an early age, leading to less production of AFt. However, excessive EMR content is not conducive to the development of strength.

**Figure 5:**
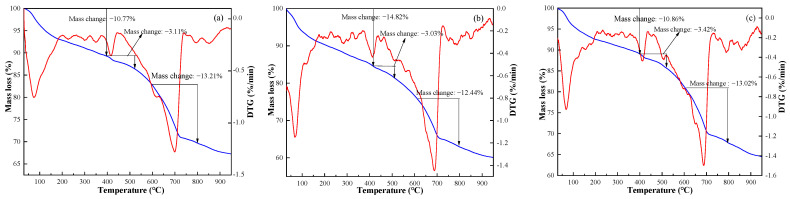
TG-DTG curves of FAC paste samples at 28 days: (**a**) FAC1, (**b**) FAC2, and (**c**) FAC3.

The TG-DTG results of FAC paste samples (FAC1, FAC2, and FAC3) hydrated at 28 days are shown in Figure 5. TG curves mainly show three weight loss ranges. The first range is 40–400 °C, corresponding to the removal of bound water in C-S-H gel, C-A-S-H gel, and AFt [28]. The corresponding mass loss of three samples at the first stage is 10.77%, 14.82%, and 10.86%, respectively, which indicates that the content of AFt and hydraulic cementitious substances is the highest in the FAC2 sample. The result is in agreement with the XRD analysis. It means that the sulfate content in FAC2 is in a suitable state, which can sufficiently activate the pozzolanic property of fly ash but will not prevent the hydration reaction. The second region (400–550 °C) is due to the thermal decomposition of calcium hydroxide [29]. The corresponding mass loss of three samples at the second stage is 3.11%, 3.03%, and 3.42%, respectively. The result shows that more Ca(OH)_2_ is consumed by the secondary hydration of fly ash to produce more cementitious substances in the FAC2 sample, which promotes strength development. The third region (600–800 °C) is related to the decomposition of CaCO_3_ and MnSO_4_. At the third stage, the corresponding mass loss is 13.21%, 12.44%, and 13.02%, respectively. The chemical reaction schemes of phase transformations of FAC paste at 30–900 °C are Schemes (6) to (10).

Ca_6_Al_2_(SO_4_)_3_(OH)_12_·26H_2_O → CaSO_4_ + CaO + Al_3_O_2_ + H_2_O (6)

C-S-H → C_3_S + C_2_S + H_2_O (7)

C-A-S-H → C-A-S + H_2_O (8)

Ca(OH)_2_ → CaO + H_2_O (9)

CaCO_3_ → CaO + CO_2_ (10)

In summary, it is thought that moderate sulfate from EMR can activate the pozzolanic activity of fly ash in the FAC2 sample, producing more cementitious substances at 3d and resulting in the highest strength. In contrast, the sulfate dosage in the FAC1 sample cannot meet the requirements of phase conversion of dissolved Si-Al to AFt, so that the 3d strength of FAC1 is lower than that of FAC2. However, the strength of FAC3 and FAC4 is even lower than FAC2. The reason could be that, as FAC3 and FAC4 samples have a higher dosage of EMR, excessive sulfate, especially the excess gypsum, can inhibit the hydration reaction of clinker, causing the cement solidification time longer [30]. There are more cementitious substances generated in the FAC2 sample than the other two samples. Fly ash can be well depolymerized in an alkaline environment. Combined with the proper sulfate effect of EMR, the hydration reaction of fly ash can be more adequate. The results can explain the reason why the FAC2 sample had the highest strength.

### 3.3. Effect of Electrolytic Manganese Residue on Microstructure of FAC

**Figure 6:**
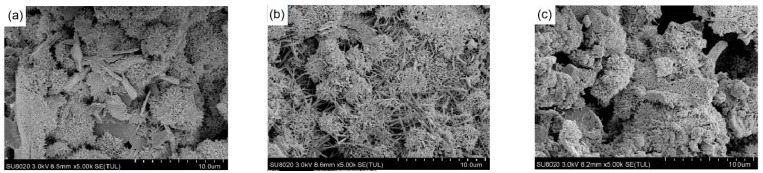
SEM images of hydration products of FAC samples at 28d: (**a**) FAC1, (**b**) FAC2, and (**c**) FAC3.

The SEM images of hydration products of three samples (FAC1, FAC2, and FAC3) hydrated at 28 days are shown in Figure 6. The amorphous gel can be seen covering the paste matrix, which is dotted with thin rod-shaped AFt and flake Ca(OH)_2_ [31,32]. The main components of amorphous gel are C-S-H gel and C-A-S-H gel. The surface of the unreacted particle is covered with fiber gels. It can be seen from Figure 6a that the surface of the FAC1 paste matrix is covered with flocculent material on which the needle-bar ettringite grows. However, the amount of ettringite is relatively less with sparse distribution, and flakier Ca(OH)_2_ is also on the surface. Previous studies have shown that excessive Ca(OH)_2_ dosage in hardened cement paste will lead to lower strength, while high content of gels will improve mechanical properties [33,34]. Figure 6b shows that the content of AFt increases significantly with serried distribution, and the spatial structure is compact, so the strength of FAC2 is higher than that of FAC1. Meanwhile, few Ca(OH)_2_ can be found in FAC2 paste. The reason is that more Ca(OH)_2_ is consumed by the pozzolanic reaction of fly ash, which leads to formation of more hydration products. With the increases of gelatinous products, the hydration products are interwoven into a network structure, making the structure more compact. It can be seen from Figure 6c that large pores appear on the matrix surface of FAC3. The amorphous gels are smaller than those in Figure 6a. It proves that the cementitious substances in FAC3 are small, and the filling of gaps between the matrix is insufficient, resulting in a looser structure and lower strength. This conforms to the results of the XRD analysis.

**Figure 7:**
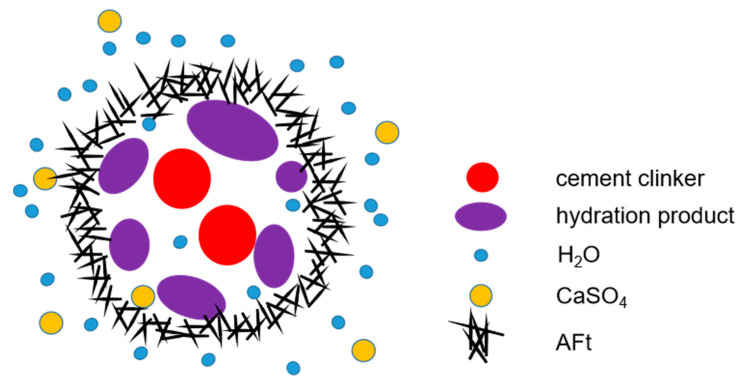
AFt covering model in the case of excessive EMR composed in the FAC.

As can be seen from Figure 6c, there are some particles and gels coated with AFt. It is known that the clinker reacts with water to form hydration products, such as tricalcium aluminate hydrate, C-S-H gel, etc. The sulfate in EMR contacts the hydration products and reacts with the fly ash to form AFt. If the sulfate content is not much, such as the FAC1 sample, the pozzolanic property of fly ash is not sufficiently activated, resulting in a low initial formation rate of AFt, which incompletely covers the surface of particles and gel. Water and sulfate salts react seamlessly with clinker and fly ash to form ettringite [12,28,35]. In other words, ettringite continues to grow. Once an excess of sulfate is added, such as FAC3 sample, much AFt is produced initially on the clinker surface, which grows and coats clinker particles and gels. Figure 7 presents an AFt covering a model in case of excess sulfate provided by EMR in the hydrated FAC3 paste. As shown in Figure 7, the dense AFt film may prevent sulfate and water from contacting the granule and prematurely terminate the formation of AFt. The volume expansion process of FAC3 is interrupted, resulting in particles less than 15 μm shown in Figure 6c. These particles may have less connection area with the matrix or even be separate from the matrix. Therefore, excessive sulfate provided by EMR is not conducive to the growth of AFt, affecting the development of strength. Cement particles clump together to form large and irregularly shaped particles. As the AFt crystal grows larger and larger, it continues to encroach on the surrounding space, resulting in expansion stress [36,37,38,39,40]. Once the expansion stress is greater than the tensile strength of AFt, the AFt film will be broken, forming fine cracks.

It is generally believed that pore structure and porosity strongly influence the mechanical properties and transport properties of cement paste [37]. The mercury intrusion test obtained the representative parameters of pore structure for three samples (FAC1, FAC2, and FAC3), and the results are shown in Table 3. It can be seen that the porosity, total pore volume, and average pore diameter of FAC2 paste are the lowest, indicating that the pore structure of the FAC2 sample is the most compact. We found that the bulk density and apparent density increased first and then decreased by comparing the three samples. Hence, more hydraulic cementitious substances are generated in the FAC2 sample [38] because the density of C-S-H gel is higher compared with other hydration products, leading to higher mechanical properties [39]. Meanwhile, it also proves that although the addition of EMR can introduce some inert substances, the optimum content of EMR can effectively improve the mechanical properties of FAC.

**Figure 8:**
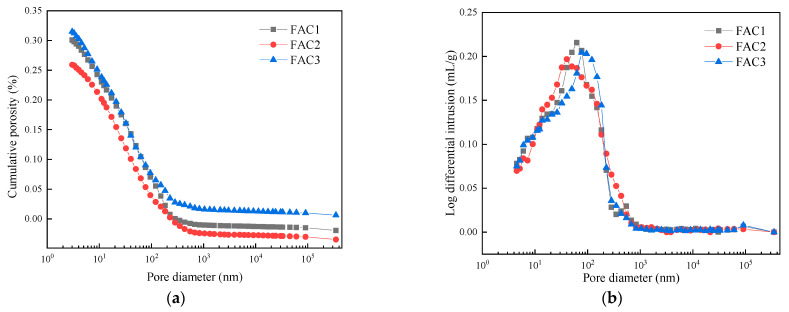
Cumulative porosity curves (**a**) and log-differential volume curves (**b**) of FAC samples hydrated at 28 days.

Critical pore diameter refers to the maximum pore level that can connect large pores, reflecting pore connectivity and permeability paths’ tortuosity [40,41]. Moreover, the critical pore diameter has the most important effect on the permeability of hardened cement paste. Figure 8 presents the cumulative porosity curves and log-differential volume curves of three samples hydrated at 28 days. The value of critical pore diameter can be obtained from the inflection point by analyzing the slope of the cumulative porosity curve in Figure 8a, and the corresponding value is 21.1, 9.1, and 22.0 nm, respectively. The smallest value of critical pore diameter is obtained by FAC2, which indicates that its pore structure is the finest [28]. Low sulfate content in FAC1 leads to the insufficient formation of AFt, which leads to a higher value of critical pore diameter than FAC2. The highest value of critical pore diameter for FAC3 is related to the cracks caused by volume expansion resulting from the excessive sulfate in FAC3. Therefore, proper EMR content can obtain a medium pore gradation, making the pore size smaller and reducing the permeability of FAC paste.

As can be seen from Figure 8b, the area surrounded by a differential curve and transverse axis represents total pore volume. The aperture corresponding to the peak value of the curve is the threshold pore diameter within a certain pore size range. It represents the pore size with the highest probability of occurrence [28,42]. Its physical meaning is that no connected pore can be formed in hardened paste when the pore size is smaller than that. It can be seen from Figure 8b that there is a gap between the total pore volume and porosity, proving that similar hydration products have been produced. Meanwhile, the threshold aperture of the FAC2 sample is the smallest, and the threshold aperture of the FAC3 sample is much higher than that of FAC1 and FAC2. It shows that the pore structure connectivity of FAC2 is weaker, resulting in lower permeability.

Table 4 shows the pore size distribution of three samples hydrated at 28 days. Previous studies show that gel pore size is less than 10 nm, the meso-capillary pore size is from 10 nm to 50 nm, and the macro-capillary pore size is more than 50 nm [43]. Among them, the macro-capillary pore is harmful to the development of strength. It can be seen from Table 4 that the porosity decreases for FAC2 when the content of EMR is 10%. Meanwhile, the content of the meso-capillary pore and macro-capillary pore decreases, the content of gel pore increases, which has a good effect on improving the macroscopic properties of FAC2, such as reducing shrinkage [44]. Many macro-capillary pores appear in FAC1 and FAC3 samples, indicating that the pore is rough and not conducive to producing a favorable void structure. FAC3 has more large pores than FAC1, mainly due to volume expansion and cracking caused by excessive sulfate, confirming the previous hypothesis. The result declares that the pores are well filled, and the pore size is small with the hydration of the FAC2 sample. In summary, the pore structure of the FAC2 sample was the best, and the strength was the highest when the EMR dosage was 10%.

^29^Si nuclear magnetic resonance (NMR) technology is often used to research the interaction around Si nuclei. As a powerful analytical tool, NMR technology has been widely used in the field of cement [45]. The chemical environment of ^29^Si is expressed by Q^n^ in silicates. Table 5 expresses the scope of the chemical shift of SiQ^n^ in ^29^Si NMR spectra. Q^n^ means that there are n silicon-oxygen tetrahedrons associated with [SiO_4_]. For example, Q^0^ means a single silicon-oxygen tetrahedron, Q^4^ represents a framework silicate structure [46,47]. In addition to the nearest atom coordination, the next-nearest atom effect also greatly influences the chemical shift. The chemical shift moves in a positive direction when the next approaching atom is an aluminum atom. Q^n^ (mAl) represents [AlO_4_] connected to [SiO_4_]. The letter “m” denotes the number of aluminum-oxygen tetrahedrons connected to the central silicon-oxygen tetrahedron. Zhang et al. [48] thought the relative number of bridging oxygen (RBO) could be calculated as follows:

(11) $$\mathrm{RBO}=\frac{1}{4}(1\times \frac{Q^{1}}{\Sigma Q^{n}}+2\times \frac{Q^{2}}{\Sigma Q^{n}}+3\times \frac{Q^{3}}{\Sigma Q^{n}}+4\times \frac{Q^{4}}{\Sigma Q^{n}})=\frac{1}{4}\times \frac{\Sigma n\cdot Q^{n}}{\Sigma Q^{n}}$$

Q^n^ means the area of the corresponding formant.

It is necessary to determine the location and attribution of the resonance peak to accurately calculate the area. The superimposed formant was divided into several peaks, then the relative area was calculated, which could characterize the polymerization degree of [SiO_4_] to some extent.

**Figure 9:**
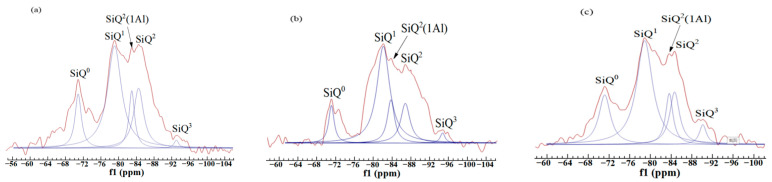
^29^Si NMR spectra of three samples hydrated at 28 days: (**a**) FAC1, (**b**) FAC2, and (**c**) FAC3.

Figure 9 shows the ^29^Si NMR spectra of three paste samples (FAC1, FAC2, and FAC3) hydrated at 28 days. The peak position, assign, relative area, and RBO value of the three samples are shown in Table 6. Q^0^ represents the tricalcium silicate and part of dicalcium silicate, which were unhydrated. Q^1^ corresponds to silicon-oxygen tetrahedron at the two ends of chain C-S-H gel. Q^2^ represents silicon-oxygen tetrahedron in the middle part of chain C-S-H gel. The assign like Q^n^ (mAl) is because of the production of C-A-S-H gel. A part of aluminum replaces the position of silicon in the reaction process. It can be seen from Figure 9 that all three samples have five resonance peaks in the spectra. They correspond to SiQ^0^, SiQ^1^, SiQ^2^(1Al), SiQ^2,^ and SiQ^3^, respectively. The Q^0^ formant of the FAC2 sample is the lowest, which indicates that the hydration of clinker is more sufficient to produce more cementitious substances. Once again, it demonstrates that appropriate sulfate can stimulate the pozzolanic activity of fly ash and promote the growth of AFt, while the sulfate content exceeding a specific range will hinder the hydration process of clinker. The RBO of the FAC2 sample presents the maximum value, indicating that the [SiO_4_] tetrahedron of the FAC2 sample has the best polymerization degree, and resulting in the highest strength [49].

### 3.4. Effect of Electrolytic Manganese Residue on Environmental Performance of FAC

Ammonia nitrogen (NH_3_-N) and heavy metals in EMR should not be ignored. In this experiment, the FAC made from EMR should meet the environmental performance evaluation index. The leaching test of EMR and FAC mortar specimens was carried out according to Chinese standard HJ/T 300-2007. Firstly, EMR and the FAC mortar specimens were immersed in PTFE bottles containing acidic buffer solution at a 1:20 solid-liquid ratio and then extracted in a rotatory machine spinning at a speed of 30 ± 2 rotations/min for 18 h. Thereafter, the percolate was analyzed by using ICP. Then, the leaching test results were compared with the national integrated sewage discharge standard GB 8978-1996, as shown in Table 7. Because the content of NH_3_-N and heavy metals in the raw EMR exceeds the standard, untreated EMR is not allowed to be directly discharged back to nature. The NH_3_-N in EMR can be effectively removed based on OH-. The heavy metal ions of EMR could be adsorbed in AFt and gels in the hydrated FAC paste [50]. The leaching test result shows that the prepared FAC has good environmental performance, and using proper content of EMR to prepare FAC is safe for the environment.

## 4. Conclusions

EMR can be used for the preparation of fly ash-based cementitious material due to the gypsum composed in EMR. The compressive strength of FAC mortar at 28 days can reach 50 MPa when the optimal content of EMR is 10%. Insufficient sulfate from EMR cannot adequately stimulate the pozzolanic activity of fly ash, while excessive content of sulfate from EMR hinders the hydration of clinker, both of which are not conducive to the early strength development of FAC. Excess sulfate from EMR will even cause cracking of FAC sample in the later stage and finally affect the strength growth.

The main hydration products of FAC are AFt, Ca(OH)_2_, C-S-H gel, C-A-S-H gel, and CaAl_2_Si_4_O_12_·4H_2_O. The content of AFt and gels is the highest in the FAC2 sample. At the same time, more Ca(OH)_2_ is consumed by the pozzolanic reaction of fly ash to promote the strength development of FAC2.

The highest RBO value of 32.60% is obtained by the FAC2 sample, indicative of the best [SiO_4_] polymerization structure. The lowest values of critical pore diameter can also be obtained by the FAC2 sample. Moreover, the pores in the FAC2 sample tend to be harmless, causing a more compact pore structure.

Leaching experiments show that Mn, Cr, Cd, As, and Pb ions in EMR could be significantly adsorbed in AFt and gels, and the NH_3_-N in EMR is effectively removed from the FAC. Thus, FAC shows good environmental performance.

## Figures and Tables

**Table 1:** Chemical composition of raw materials (%).

Oxides	SiO_2_	Al_2_O_3_	Fe_2_O_3_	CaO	MgO	MnO	SO_3_	LOI
EMR	29.63	8.32	5.41	14.82	1.79	2.02	29.54	8.47
FA	47.38	37.09	8.02	3.12	0.34	0.05	1.35	2.63
Clinker	18.6	3.81	3.25	64.85	2.89	0.34	0.43	4.14

**Table 2:** Proportions of FAC samples (wt%).

Samples	Clinker	EMR	FA	SO_3_	Fineness (m^2^/kg)
FAC0	55	0	45	0.8	377
FAC1	55	5	40	2.3	380
FAC2	55	10	35	3.1	385
FAC3	55	15	30	4.2	376
FAC4	55	20	25	5.4	373

**Table 3:** The representative parameters of pore structure of FAC paste hydrated at 28 days.

Samples	BET (m^2^/g)	Total Pore Volume (mg/L)	Average Pore Diameter (nm)	Porosity (%)	Bulk Density (g/cm^3^)	Apparent Density (g/cm^3^)
FAC1	231.00	0.31	17.6	40.32	1.30	2.22
FAC2	230.00	0.29	15.8	39.44	1.34	2.24
FAC3	232.00	0.32	18.4	41.76	1.30	2.19

**Table 4:** Pore size distribution of FAC samples hydrated at 28 days.

Samples	Porosity (%)	Pore Size Distribution (%)		
		<10 nm	10–50 nm	>50 nm
FAC1	40.32	7.91	16.17	16.24
FAC2	39.44	9.78	15.11	14.55
FAC3	41.76	9.15	13.95	18.66

**Table 5:** ^29^Si NMR chemical shift of SiQ^n^ in solid silicates.

Symbol	SiQ^0^	SiQ^1^	SiQ^2^	SiQ^3^	SiQ^4^
Chemical shift (ppm)	−68–−76	−76–−82	−82–−88	−88–−98	−98–−129

**Table 6:** The RBO values of FAC samples hydrated at 28 days.

Samples	Peak Position (ppm)	Assign	Relative	RBO Value
FAC1	−71.29	SiQ^0^	23.83	30.99%
	−79.63	SiQ^1^	100	
	−84.80	SiQ^2^(1Al)	22.27	
	−85.15	SiQ^2^	41.80	
	−93.81	SiQ^3^	2.73	
FAC2	−71.45	SiQ^0^	16.31	32.60%
	−81.37	SiQ^1^	100	
	−83.97	SiQ^2^(1Al)	29.08	
	−87.00	SiQ^2^	34.75	
	−94.84	SiQ^3^	4.26	
FAC3	−71.54	SiQ^0^	35.02	29.58%
	−79.42	SiQ^1^	100	
	−84.29	SiQ^2^(1Al)	22.96	
	−85.34	SiQ^2^	31.13	
	−90.91	SiQ^3^	8.56	

**Table 7:** Results of leaching tests (mg/L).

Samples	Mn	Cr	Cd	As	Pb	NH_3_-N
EMR	1168	0.205	0.064	0.158	0.293	24.53
FAC1	<0.001	<0.001	<0.001	<0.001	0.001	0.26
FAC2	<0.001	0.001	<0.001	<0.001	0.003	0.35
FAC3	<0.001	0.002	<0.001	<0.001	0.005	0.52
GB/T 8978-1996	2.0	1.5	0.1	0.5	1.0	15

## Data Availability

Data sharing is not applicable to this article.

## Supplementary Materials

The following are available online at https://www.mdpi.com/article/10.3390/ma14227047/s1, Figure S1: XRD pattern of calcium carbide slag, Figure S2: XRD pattern of fly ash.
