# Supplementary material for: Effect of Electrolytic Manganese Residue in Fly Ash-Based Cementitious Material: Hydration Behavior and Microstructure
Source: Materials (Basel). 2021 Nov 20;14(22):7047. doi: 10.3390/ma14227047 (PMC8621800; doi:10.3390/ma14227047)
Supplement: Supplementary file 1 [file materials-14-07047-s001.zip › materials-1380344-supplementary.pdf]

materials

MDPI

Supplementary material

Effect of Electrolytic Manganese Residue in Fly Ash-Based Cementitious Material: Hydration Behavior and Microstructure

Yaguang Wang [1], Na Zhang [2,*], Yongyu Ren [1], Yingtang Xu [1] and Xiaoming Liu [1,*]

[1] State Key Laboratory of Advanced Metallurgy, School of Metallurgical and Ecological Engineering, University of Science and Technology Beijing, Beijing 100083, China; wangyg@xs.ustb.edu.cn (Y.W.); ren142517@163.com (Y.R.); yingtangx@163.com (Y.X.)

[2] Beijing Key Laboratory of Materials Utilization of Nonmetallic Minerals and Solid Wastes, National Laboratory of Mineral Materials, School of Materials Science and Technology, China University of Geosciences, Beijing 100083, China

* Correspondence: nazhang@cugb.edu.cn (N.Z.); liuxm@ustb.edu.cn (X.L.)

Citation: Wang, Y.; Zhang, N.; Ren, Y.; Xu, Y.; Liu, X. Effect of Electrolytic Manganese Residue in Fly Ash-Based Cementitious Material: Hydration Behavior and Microstructure. *Materials* **2021**, *14*, 7047. https://doi.org/10.3390/ma14227047

Academic Editors: Alex Kondratiev, Dmitry Valeev and Neven Ukrainczyk

Received: 30 August 2021
Accepted: 16 November 2021
Published: 20 November 2021

Publisher's Note: MDPI stays neutral with regard to jurisdictional claims in published maps and institutional affiliations.

Copyright: © 2021 by the authors. Licensee MDPI, Basel, Switzerland. This article is an open access article distributed under the terms and conditions of the Creative Commons Attribution (CC BY) license (http://creativecommons.org/licenses/by/4.0/).

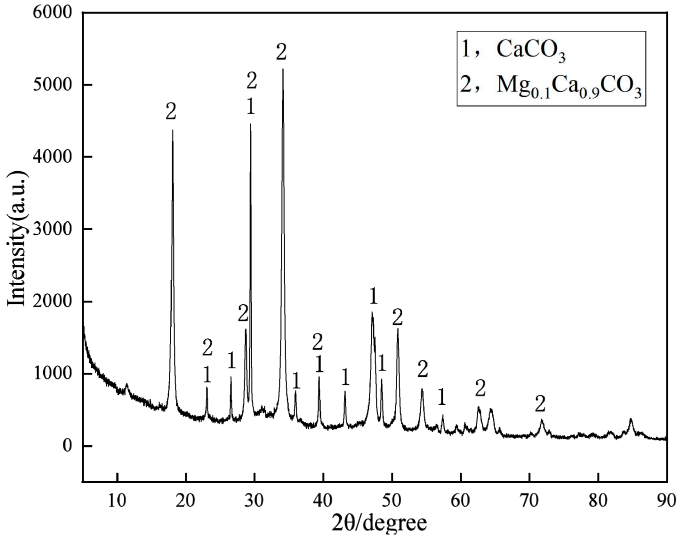

Figure S1. XRD pattern of calcium carbide slag.

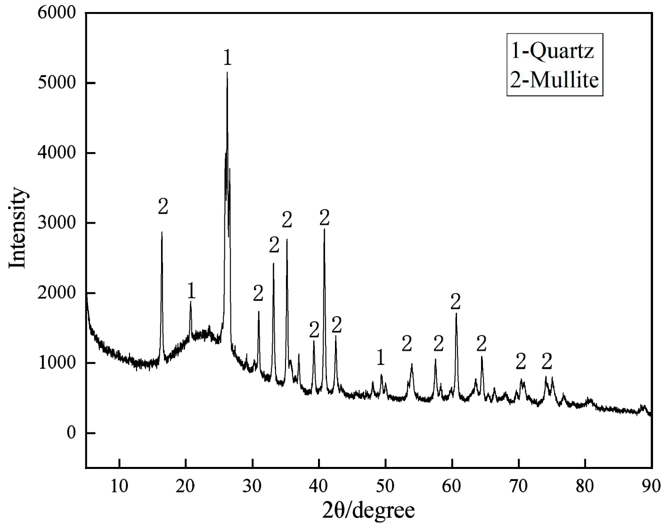

Figure S2. XRD pattern of fly ash.
